# Incidence of hand and wrist disorders in primary care: a retrospective cohort study

**DOI:** 10.3399/BJGPO.2023.0240

**Published:** 2024-10-02

**Authors:** Patrick Krastman, Evelien IT de Schepper, Patrick JE Bindels, Sita MA Bierma-Zeinstra, Gerald Kraan, Jos Runhaar

**Affiliations:** 1 Department of General Practice, Erasmus MC University Medical Center Rotterdam, Rotterdam, The Netherlands; 2 Department of Orthopedics & Sports Medicine, Erasmus MC University Medical Center Rotterdam, Rotterdam, The Netherlands; 3 Department of Orthopedic Surgery, Reinier de Graaf Groep, Delft, The Netherlands

**Keywords:** incidence, hand, wrist, primary health care

## Abstract

**Background:**

The incidence of different types of hand and wrist disorders in primary care is unknown since there are no specific encodings for it.

**Aim:**

To determine the overall incidence and the incidence of specific types of hand and wrist disorders in primary care.

**Design & setting:**

A retrospective cohort study was undertaken, using a healthcare registration database from Dutch general practice, which contains medical records of more than 200 000 patients, and included approximately 25% of the population of the area of Rotterdam in The Netherlands.

**Method:**

Patients aged ≥18 years with a new diagnosis of hand or wrist disorder from 1 January 2015–31 December 2019 were extracted using a search algorithm based on International Classification of Primary Care (ICPC) coding and search terms in free text.

**Results:**

The mean incidence over the study period of a hand disorder was 5.9 per 1000 persons-years and of a wrist disorder 0.3 per 1000 persons-years. The incidence of trigger finger or thumb, hand or finger fracture, tendon or ligament tendinopathy, mallet finger, and hand or finger ligament injury were 3 (95% confidence interval [CI] = 2.69 to 3.15), 1 (95% CI = 1.03 to 1.33), 1 (95% CI = 0.98 to 1.28), 0.6 (95% CI = 0.48 to 0.69), and 0.1 (95% CI = 0.06 to 0.14) per 1000 persons-years, respectively. The incidence of a wrist fracture and ligament injury were 0.2 (95% CI = 0.13 to 0.25) and 0.1 (95% CI = 0.04 to 0.12) per 1000 persons-years, respectively.

**Conclusion:**

There is a large difference between the number of patients presenting to the GP with hand and wrist complaints and the number of hand and wrist diagnoses reported in the medical files. Introducing specific ICPC codes for different types of hand and wrist disorders could (potentially) lead to a more accurate registration of a diagnosis and determination of the incidence figures.

## How this fits in

Current epidemiologic research on hand and wrist disorders in primary care is largely limited to complaints and general codified data, obtained from primary care databases. To the authors’ knowledge, this is the first study to provide an insight in the incidence of hand and wrist disorders in primary care. Based on the incidence figures described in this study, there is a large difference between the number of patients presenting to the GP with hand and wrist complaints and the number of hand and wrist diagnoses reported in the medical files of the GP. GPs assess more patients with hand than wrist disorders per year, with a trigger finger diagnosed most often, and a wrist fracture or ligament injury of the hand and wrist was seen sporadically.

## Introduction

Hand and wrist disorders are common. Depending on the healthcare organisation, patients with hand and wrist complaints first report to a general practice or, in urgent situations, to an emergency department of a hospital. A diagnosis is based on the patient’s history and complaints, physical examination and, if necessary, imaging. If the clinician is unable to make a diagnosis, a wait-and-see policy or referral to a hand and wrist specialist is a possible next step in the management.

Current epidemiologic research on hand and wrist disorders in primary care is largely limited to complaints and general codified data, obtained from primary care databases. In Dutch general practice, as defined by the International Classification of Primary Care (ICPC) codes, the incidence of hand and finger complaints is approximately 24.1 new episodes per 1000 patients per year and that of wrist complaints is about 10.3 episodes per 1000 patients per year.^1^ Although some hand and wrist disorders have a specific ICPC code, these disorders may be described and coded under the general hand and wrist complaints, possibly because the GP cannot diagnose the specific disorder at the first visit. Moreover, multiple hand and wrist disorders do not have a suitable unique ICPC code. Also, complaint diagnoses may not be adequately updated or recoded to a specific diagnosis by the GP after disease progression or a change in the final diagnosis.^2^ For this reason, the exact incidence of different types of hand and wrist disorders (for example, fractures, ligament disorders) in primary care are still unknown. Accurate and up-to-date knowledge of incidence figures of hand and wrist disorders are an essential step in preparing services for the delivery of future hand and wrist health care (at the right place) and needed for clinicians, researchers, and healthcare policymakers in order to help to facilitate adequate allocation of healthcare services. Incidence figures of hand and wrist disorders in primary care could influence GPs during the diagnostic process and managing patients with these complaints. The purpose of this study was to determine the overall incidence and the incidence of different types of hand and wrist disorders diagnosed in Dutch primary care.

## Method

### Study design

A cohort study was performed using the Rijnmond Primary Care database (RPCD). The RPCD is a region-specific product of the Integrated Primary Care Information (IPCI) database, supervised by the Department of General Practice of the Erasmus University Medical Center (Erasmus MC), Rotterdam, The Netherlands. More information about the IPCI database has been d1escribed in detail elsewhere.^3^ The RPCD is a longitudinal observational dynamic database containing medical records of more than 200 000 patients in and around the Dutch city of Rotterdam. These pseudonymised medical records contain demographics, medical notes (free text), diagnoses (including ICPC codes), referrals, imaging results, and specialists’ letters that are routinely collected by GPs. The database included approximately 25% of the population of the area of Rotterdam, equally distributed across the region and including neighbourhoods with different socioeconomic and migration levels.

### Study cohort

The study population consisted of adults aged ≥18 years with a new episode of hand or wrist disorder between 1 January 2015 and 31 December 2019. The number of patients aged ≥18 years in the RPCD ranged from 185 093 in 2015 to 254 345 in 2019. The diagnosis was considered new if the patient had not been diagnosed with a hand or wrist disorder in the preceding 12 months, irrespective of the study period. For the same type of disorder, patients could be included in the study population more than once if there was >12 months between the initial diagnosis and subsequent consultations for a hand or wrist disorder.

Final diagnoses of a hand or wrist disorder were identified using complaint and specific ICPC coding and with supporting keywords in the free text.^4^ In a pilot phase, a small sample of patients with hand and wrist complaints and disorders were selected from the database and their medical files were screened for terms used by the GP to inform our search terms. Patients were considered a ‘potential hand or wrist disorder case’ if they received one of the described ICPC codes, in combination with the (Dutch) keywords in the free text, letters, or diagnosis text.^3^ By combining various ICPC codes and associated keywords, multiple algorithms were composed for each hand and wrist disorder. The algorithm excluded hits that were combined with terms of negation (for example, 'not' or 'no'). Supplementary Table S1 describes the examined hand and wrist disorders with the corresponding ICPC codes and associated keywords. During a first consultation, the GP can initially register the health problem with a complaint or symptom code (hand or finger: ICPC L12; and wrist: ICPC L11) and during the follow-up of the patient, rename or recode the complaint in a more specific diagnosis code, see Supplementary Table S1. Several hand and wrist disorders were deemed out of scope of this study: carpal tunnel syndrome (CTS), distal radius fracture, osteoarthritis, ganglia, and dupuytren contracture, since (1) incidence figures of CTS and ganglion are already known, (2) the focus of this study is not on chronic conditions, such as osteoarthritis and dupuytren contracture, and (3) the distal radius is not considered to be part of the wrist.

### Data extraction

If the number of potential cases for a specific disorder was <200 (over the study period) after running the search algorithm for this specific disorder, all files were reviewed (PK) to obtain true cases and incidence numbers. If the number of potential cases for a specific disorder after running the search algorithm was >200 (over the study period), 200 random medical files from potential cases were manually reviewed (PK) to obtain the percentage of true cases (%TC) per disorder. The medical files were examined from the consultation date of the initial diagnosis, up to 6 months after the first consultation. Potential cases were a true case if the GP defined the consultation as a type of hand or wrist disorder. Unclear cases were also assessed by a hand surgeon (GK), and final decisions were based on consensus. For each patient, year of birth and sex were additionally extracted.

### Statistics

Incidence numbers per study year, as obtained by the search algorithm, were multiplied with the %TC to obtain annual incidence estimates in case of >200 potential cases. If the potential cases of an disorder involved <200 medical files, the annual incidence numbers were determined, without having to calculate a %TC first. The 95% confidence intervals (CIs) were calculated from the incidence, using Poisson distribution. The annual incidence of any hand and of any wrist disorder per 1000 persons-years was also subdivided for males and females, and for age categories 18–40 years, 41–60 years, and ≥61 years. R Studio was used for Poisson distributions.

## Results

### Hand

The total number of patients per year with a hand disorder was 1148, 1221, 1166, 1297, and 1310 from 2015–2019, respectively. At diagnosis, the mean age was 53 (standard deviation [SD] 17.8) years. Of the included hand cases, 61% (*n* = 3735) involved female and 39% (*n* = 2407) male. Over the years, with regard to hand disorders, there has been a minimal change (dispersion) in the distribution over sex, age, and incidence. See Table 1 for more detailed information about baseline characteristics of included patients with hand disorders over the study period.

**Table 1.** Baseline characteristics of included hand and wrist disorders from 2015–2019

	2015–2019	2015	2016	2017	2018	2019
**Hand**						
Total, *n* (mean/year)	6142 (1228)	1148	1221	1166	1297	1310
Age, years, *n* (%)						
18–40	1325 (21.6)	248 (21.6)	278 (22.8)	258 (22.1)	261 (20.1)	280 (21.4)
41–60	2401 (39.1)	457 (39.8)	486 (39.8)	462 (39.6)	514 (39.6)	482 (36.8)
≥60	2416 (39.3)	443 (38.6)	457 (37.4)	446 (38.3)	522 (40.2)	548 (41.8)
Sex, *n* (%)						
Female	3735 (60.8)	701 (61.1)	750 (61.4)	715 (61.3)	802 (61.8)	767 (58.5)
Male	2407 (39.2)	447 (38.9)	471 (38.6)	451 (38.7)	495 (38.2)	543 (41.5)
**Wrist**						
Total, *n* (mean/year)	263 (53)	47	52	57	47	60
Age, years, *n* (%)						
18–40	102 (38.8)	14 (29.8)	21 (40.4)	24 (42.1)	20 (42.6)	23 (38.3)
41–60	96 (36.5)	22 (46.8)	18 (34.6)	21 (36.8)	18 (38.3)	17 (28.3)
≥60	65 (24.7)	11 (23.4)	13 (25.0)	12 (21.1)	9 (19.1)	20 (33.3)
Sex, *n* (%)						
Female	140 (53.2)	29 (61.7)	27 (51.9)	31 (54.4)	22 (46.8)	31 (51.7)
Male	123 (46.8)	18 (38.3)	25 (48.1)	26 (45.6)	25 (53.2)	29 (48.3)

**Figure 1.**
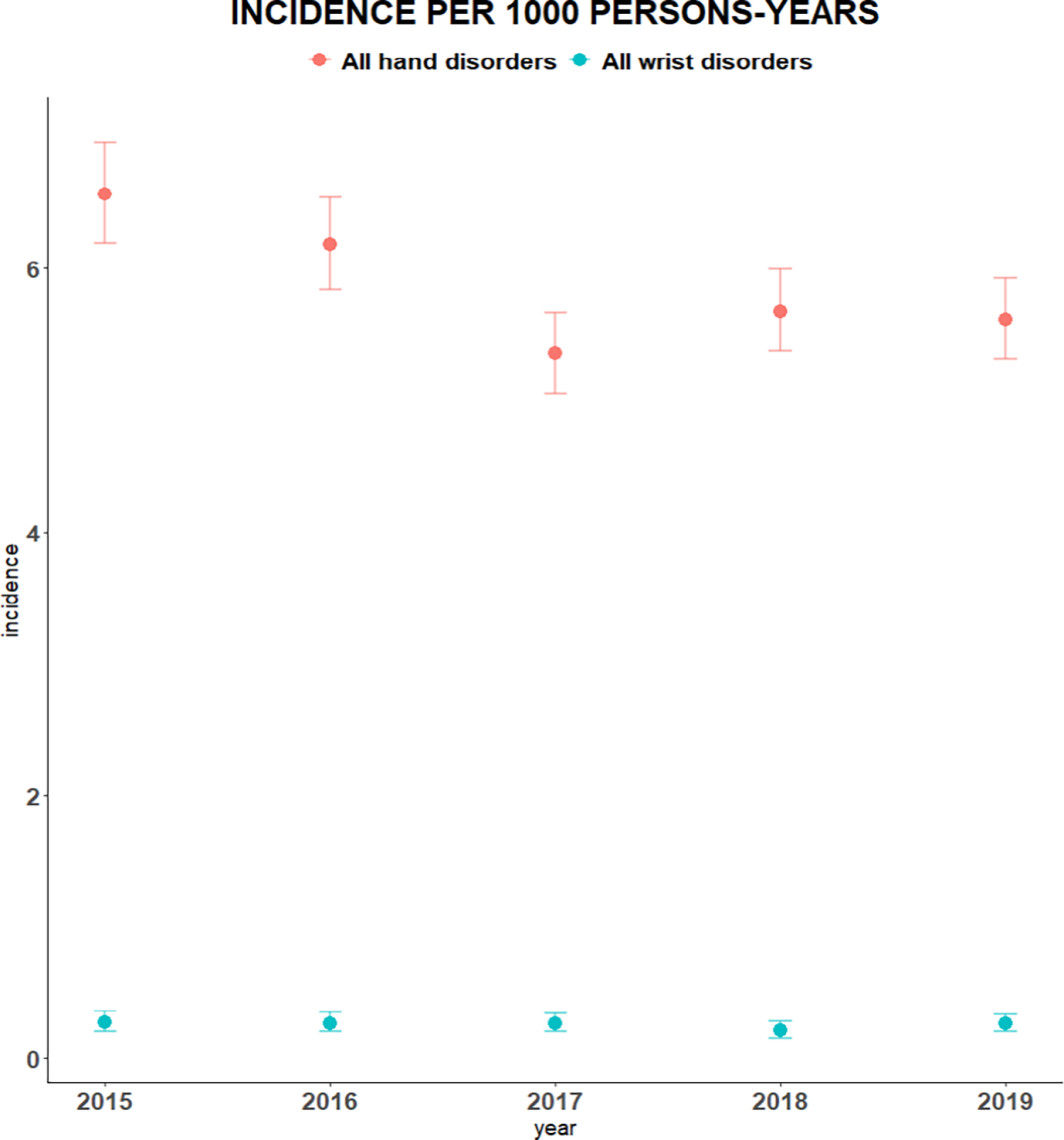
The annual incidence of hand and wrist disorders per 1000 persons-years

The mean incidence of a hand disorder over the study period was 5.9 (95% CI = 5.6 to 6.2) per 1000 persons-years. The annual incidence of hand disorders per 1000 persons-years is displayed in Figure 1.

**Figure 2.**
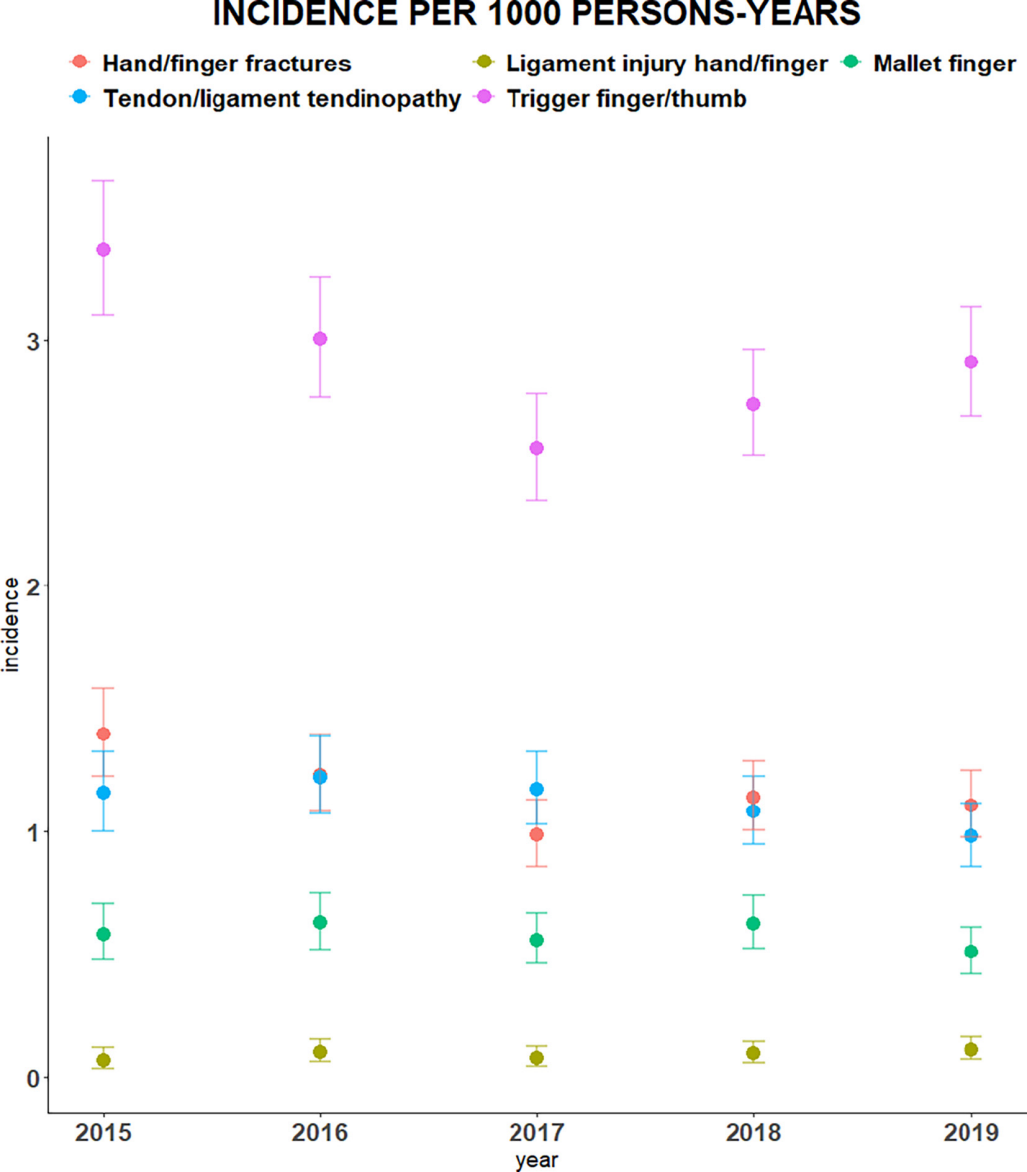
Annual incidence of different types of hand disorders per 1000 persons-years

In Figure 2 the annual incidence of different types of hand disorders (that is, trigger finger or thumb, hand or finger fracture, mallet finger, tendon or ligament tendinopathy, and hand or finger ligament injury) per 1000 persons-years is displayed.

Supplementary Table S2 shows the mean incidence of different types of hand disorders over the study period. The incidence of trigger finger or thumb, hand or finger fracture, tendon or ligament tendinopathy, mallet finger, and hand or finger ligament were 3 (95% CI = 2.69 to 3.15), 1 (95% CI = 1.03 to 1.33), 1 (95% CI = 0.98 to 1.28), 0.6 (95% CI = 0.48 to 0.69), and 0.1 (95% CI = 0.06 to 0.14) per 1000 persons-years, respectively.

### Wrist

The number of patients per year with a wrist disorder was 47, 52, 57, 47, and 60 per 1000 persons-years from 2015–2019, respectively. At diagnosis, the mean age was 48 (SD 19.3) years. Of the wrist cases, 53% (*n* = 140) were female and 47% (*n* = 123) male. Over the years, with regard to wrist disorders, there has been minimal change (dispersion) in the distribution over sex, age, and incidence. See Table 1 for more detailed information about baseline characteristics of included patients with wrist disorders over the study period. The incidence of wrist disorders over the study period was 0.3 (95% CI = 0.2 to 0.3) per 1000 persons-years. The annual incidence of wrist disorders per 1000 persons-years is displayed in Figure 1, which shows a relatively stable incidence throughout the study period.

**Figure 3.**
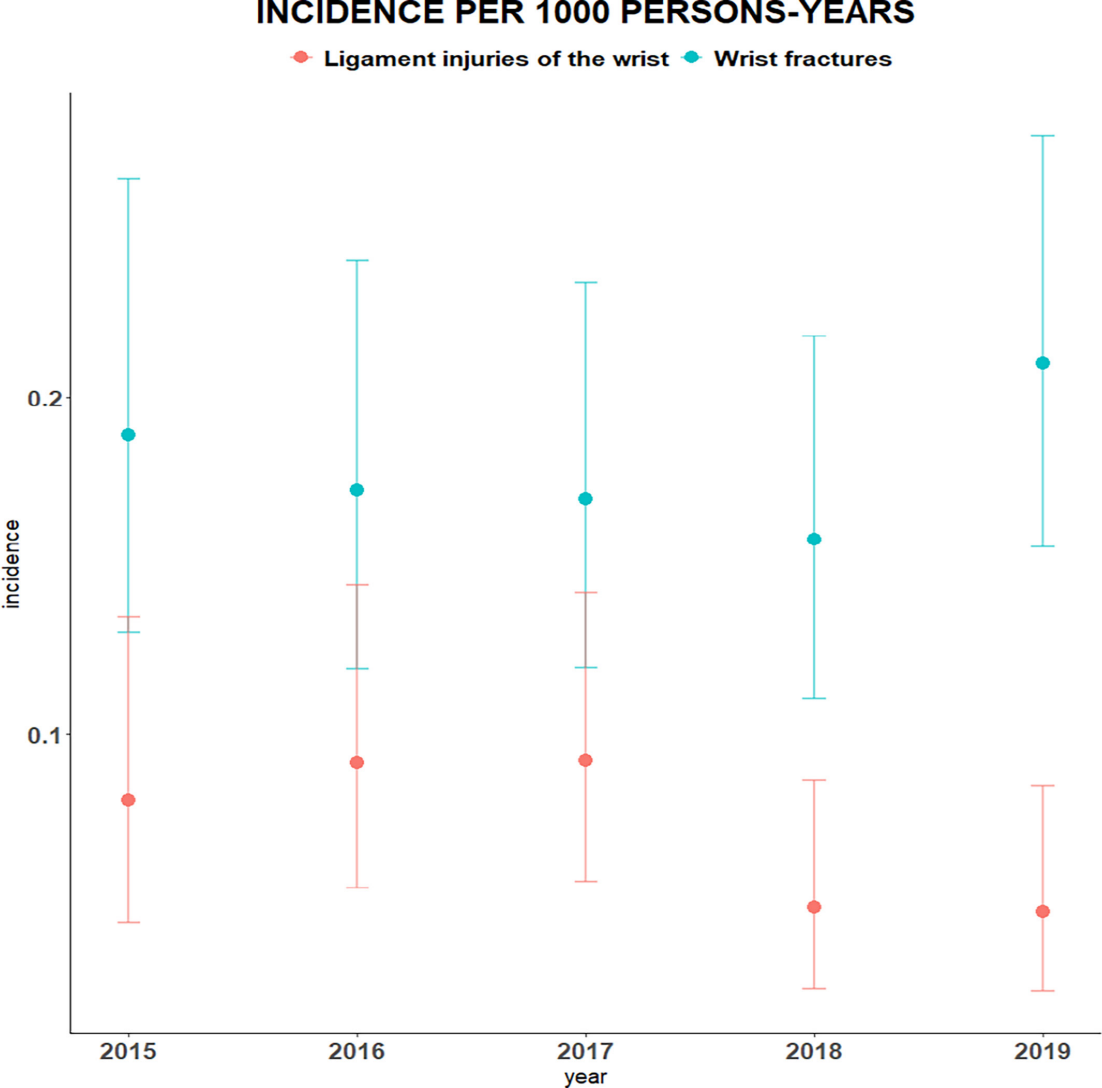
Annual incidence of different types of wrist disorders per 1000 persons-years

Figure 3 displays the annual incidence of wrist disorders (namely bone fractures and ligament injury) per 1000 persons-years.

Supplementary Table S2 shows the mean incidence of two different types of wrist disorders over the study period. The incidence of a wrist fracture and ligament injury were 0.2 (95% CI = 0.13 to 0.25) and 0.1 (95% CI = 0.04 to 0.12) per 1000 persons-years, respectively.

## Discussion

### Summary

The main findings of this study were that a GP assesses more patients with hand than wrist disorders per year, with a trigger finger diagnosed most often and a wrist fracture or ligament injury of the hand and wrist only sporadically. The incidence of each condition per 1000 persons-years were as follows: trigger finger or thumb 3 (95% CI = 2.69 to 3.15); hand or finger fracture 1 (95% CI = 1.03 to 1.33); tendon or ligament tendinopathy 1 (95% CI = 0.98 to 1.28); mallet finger 0.6 (95% CI = 0.48 to 0.69); and hand or finger ligament injury 0.1 (0.06 to 0.14). The incidence of a wrist fracture and ligament injury were 0.2 (95% CI = 0.13 to 0.25) and 0.1 (95% CI = 0.04 to 0.12) per 1000 persons-years, respectively.

### Strengths and limitations

The RPCD population is representative for the general Dutch population in terms of age and sex.^5^ The geographical spread is limited to a specific Dutch region, but GP practices are located both in urban and non-urban areas. There is no selection on type of health insurance or social economic status of patients in RPCD.^3^ The use of registration data can be seen as a strength of this study, as all Dutch citizens are registered with a GP practice. The majority of all patients with hand and wrist complaints first report to and are assessed by their GP (gatekeeper), since going directly to an emergency department first is discouraged. To limit the possible underestimation of the overall incidence because of limited medical notes or non-uniform ICPC coding, multiple ICPC codes and free-text terms to identify patients with hand and wrist disorders were used.^6^ The strength of this study is that the diagnoses that were coded with a general hand or wrist complaint code by the GP, were also assessed for a specific diagnosis.

In The Netherlands, patients have direct access to a paramedical professional, without requiring a referral from their GP. Therefore, it is possible that patients with hand or wrist disorders have not initially consulted their GP, potentially resulting in an underestimation of the incidence figures in this study. Other causes of a potential underestimation of the incidence figures and possible explanations for the discrepancy between complaints and a diagnosis could be that: (1) many complaints are self-limiting, which supports a wait-and-see policy; (2) of a few hand and wrist disorders, without specific ICPC codes (for example, luxation finger, osteoarthritis of the hand and wrist), the incidence figures have not been determined; (3) GPs lack diagnostic expertise and tools for diagnosing hand and wrist disorders; (4) GPs receive no or incomplete feedback from secondary care organisations; and (5) GPs insufficiently report or code the definitive disorders in the medical file.^7–9^

Since this study was dependent on GP medical records, the incidence should be considered as the incidence of registered consultations only.

### Comparison with existing literature

There is a lack of data on specific hand and wrist disorders in primary care. Therefore, a comparison with existing data from primary care studies could not be performed.

According to Nielen *et al*, in Dutch general practice, the incidence of hand and finger complaints is approximately 24 new episodes per 1000 patients per year and that of wrist complaints is about 10 episodes per 1000 patients per year.^1^ Based on the incidence figures described in this study, there is a large difference between the number of patients presenting to the GP with hand and wrist complaints and the number of hand and wrist diagnosis reported in the medical files of the GP. The incidence figures of different types of hand and wrist disorders in primary and secondary care are scarce and mostly unknown.^10–15^ A population-based cohort study in the US (Olmsted County, Minnesota) described an incidence rate of 0.33 acute traumatic tendon injuries in the hand and wrist per 1000 person-years.^15^ This study determined an incidence rate of 0.2 tendon injuries in the hand and wrist per 1000 person-years. One large retrospective study of mallet fingers at a UK hospital’s emergency department reported an incidence of 0.99 per 1000 patient-years.^16^ In this study, all patients with musculoskeletal injuries are either directly admitted to the ward or referred for follow-up in the orthopaedic trauma unit outpatient clinics. This may explain the difference in mean incidence compared with the current study, where a mean incidence of 0.6 per 1000 persons-years was found in patients initially assessed by a GP.

### Implications for research and practice

There is a large difference between the number of patients presenting to a GP with hand and wrist complaints and the number of hand and wrist diagnosis reported in the GP’s medical files. As mentioned above, several causes could explain this observation. To determine the incidence of different types of hand and wrist disorders in primary care, adequate registration is essential. Accurate and up-to-date knowledge of incidence figures of hand and wrist disorders are: (1) an essential step in preparing services for the delivery of future hand and wrist health care (at the right place); (2) needed for clinicians and researchers to better study trends over time and associations of risk factors; and (3) essential for GPs to understand the probability of a certain diagnosis in clinical practice and to manage patients more adequately.

The introduction of specific ICPC codes for different types of hand and wrist disorders, such as for scaphoid fractures and de Quervain's tenosynovitis, could (potentially) lead to a more accurate registration of these diagnoses and determination of the incidence figures. During the initial diagnostic process (that is, first consultation), the GP might initially register the health problem with a general hand and wrist complaint code and over time (for example, after medical specialist diagnoses from return letters) rename or recode the complaint in a more specific diagnosis. Further research is needed to better understand the registration and coding behaviour of GPs.

In conclusion, GPs assess more patients with hand than wrist disorders per year, with a trigger finger diagnosed most often and a wrist fracture or ligament injury of the hand and wrist only sporadically. To determine the incidence of different types of hand and wrist disorders in primary care, adequate registration is essential. Incidence figures are crucial for GPs, to understand the probability of a certain diagnosis in clinical practice, and to manage patients more adequately.

## Data

All datasets can be made available to readers on request.
